# Sericin cocoon bio-compatibilizer for reactive blending of thermoplastic cassava starch

**DOI:** 10.1038/s41598-021-99417-3

**Published:** 2021-10-07

**Authors:** Thanongsak Chaiyaso, Pornchai Rachtanapun, Nanthicha Thajai, Krittameth Kiattipornpithak, Pensak Jantrawut, Warintorn Ruksiriwanich, Phisit Seesuriyachan, Noppol Leksawasdi, Yuthana Phimolsiripol, Charin Techapun, Sarana Rose Sommano, Toshiaki Ougizawa, Kamon Yakul, Kittisak Jantanasakulwong

**Affiliations:** 1 School of Agro-Industry, Faculty of Agro-Industry, Chiang Mai University, Mae Hia, Muang, Chiang Mai Thailand; 2 Cluster of Agro Bio-Circular-Green Industry, Faculty of Agro-Industry, Chiang Mai University, Mae Hia, Muang, Chiang Mai Thailand; 3 Center of Excellence in Materials Science and Technology, Faculty of Science, Chiang Mai University, Mae Hia, Muang, Chiang Mai Thailand; 4 Faculty of Science, Chiang Mai University, Mae Hia, Muang, Chiang Mai Thailand; 5 Department of Pharmaceutical Sciences, Faculty of Pharmacy, Chiang Mai University, Mae Hia, Muang, Chiang Mai Thailand; 6 Plant Bioactive Compound Laboratory (BAC), Department of Plant and Soil Sciences, Faculty of Agriculture, Chiang Mai University, Mae Hia, Muang, Chiang Mai Thailand; 7 Department of Chemistry and Materials Science, Tokyo Institute of Technology, Meguro-ku, Tokyo, Japan

**Keywords:** Biochemistry, Materials science

## Abstract

Cassava starch was blended with glycerol to prepare thermoplastic starch (TPS). Thermoplastic starch was premixed with sericin (TPSS) by solution mixing and then melt-blended with polyethylene grafted maleic anhydride (PEMAH). The effect of sericin on the mechanical properties, morphology, thermal properties, rheology, and reaction mechanism was investigated. The tensile strength and elongation at break of the TPSS10/PEMAH blend were improved to 12.2 MPa and 100.4%, respectively. The TPS/PEMAH morphology presented polyethylene grafted maleic anhydride particles (2 μm) dispersed in the thermoplastic starch matrix, which decreased in size to approximately 200 nm when 5% sericin was used. The melting temperature of polyethylene grafted maleic anhydride (121 °C) decreased to 111 °C because of the small crystal size of the polyethylene grafted maleic anhydride phase. The viscosity of TPS/PEMAH increased with increasing sericin content because of the chain extension. Fourier-transform infrared spectroscopy confirmed the reaction between the amino groups of sericin and the maleic anhydride groups of polyethylene grafted maleic anhydride. This reaction reduced the interfacial tension between thermoplastic starch and polyethylene grafted maleic anhydride, which improved the compatibility, mechanical properties, and morphology of the blend.

## Introduction

Thermoplastic starch (TPS) is prepared using a plasticizer with a high temperature and shear condition to form an amorphous structure^1^ that provides a processing method similar to that of petroleum polymers. A suitable plasticizer forms hydrogen bonds between the hydroxyl groups of starch and glycerol^2^. TPS is prepared by melt-mixing granule starch and plasticizers and then converted using various methods, such as blowing^1^, casting^3^, extrusion^4^, internal mixer^5^, and two roll mills^6^. TPS produced from biomaterials are non-toxic^7^ and biodegradable^8^. The starch present in TPS could be efficiently biodegraded by amylase enzymes under mild conditions^9^ or subjected to a specialized enzyme potentially developed for starch digestion under high salt conditions^10^ to assess its biodegradability. Therefore, various raw materials of starch can be used to prepare TPS, such as cassava^11^, mung bean^12^, potato^13^, and corn^14,15^. Some plasticizers are used to plasticize starch, such as glycerol^16^, sunflower oil^17^, and soybean oil^18^.

Sericin has attracted attention because of its healing ability and is extracted from cocoon^19^ with fibroin using various methods^20,21^. It is used as an adhesive biopolymer for pharmaceutical, food, and cosmetic applications as a high-value additive. Sericin is a hydrophilic material that has excellent compatibility with other hydrophilic biopolymers, such as starch, polyvinyl alcohol (PVA), and alginate. The structure of serin contains many amino groups^22^ with a reaction capability to several reactive groups, such as maleic anhydride groups^23^ and epoxy groups^24^.

Reactive blending is an effective process for improving the properties of polymer blends^25,26^. The properties of suitable reactive biomaterials, such as carboxymethyl cellulose (CMC)^27–29^, chitosan (CTS)^5,30^, carboxymethyl chitosan^31^, carboxymethyl bacterial cellulose^32,33^, polysaccharides from rich bran^34,35^, pectin^36–38^, and keratin^39^, have been investigated for reactive blending. The improvement of mechanical properties of TPS blend with poly(butylene adipate-co-terephthalate)^40^, epoxidized natural rubber^5^, and PEMAH^41^ were investigated. Moreover, CMC- and chitosan-modified TPS for reactive blending have also been previously reported^5,14,41^. CMC-modified TPS has a low reaction activity and melt processing ability, while the acidification water dissolution of chitosan induces a brown color and acid evaporation during the melt process of TPS. However, the mechanical property improvement of TPS blend with PEMAH and sericin compatibilizer, has not been reported thus far.

Therefore, aim of this research is to develop high mechanical properties TPS using reactive melt blending of cassava starch, glycerol, sericin and PEMAH. Cassava starch was melt-blended with glycerol to prepare the TPS. TPS was modified with sericin (TPSS) for blending with polyethylene grafted maleic anhydride (PEMAH). Sericin was extracted from cocoons and incorporated into the TPS as a compatibilizer by solution mixing, and the effect of sericin on the mechanical properties, morphology, surface tension, thermal properties, rheology, and reaction mechanism was investigated to provide high tensile strength TPS for packaging, agriculture, and medical applications.

## Results and discussions

**Figure 1:**
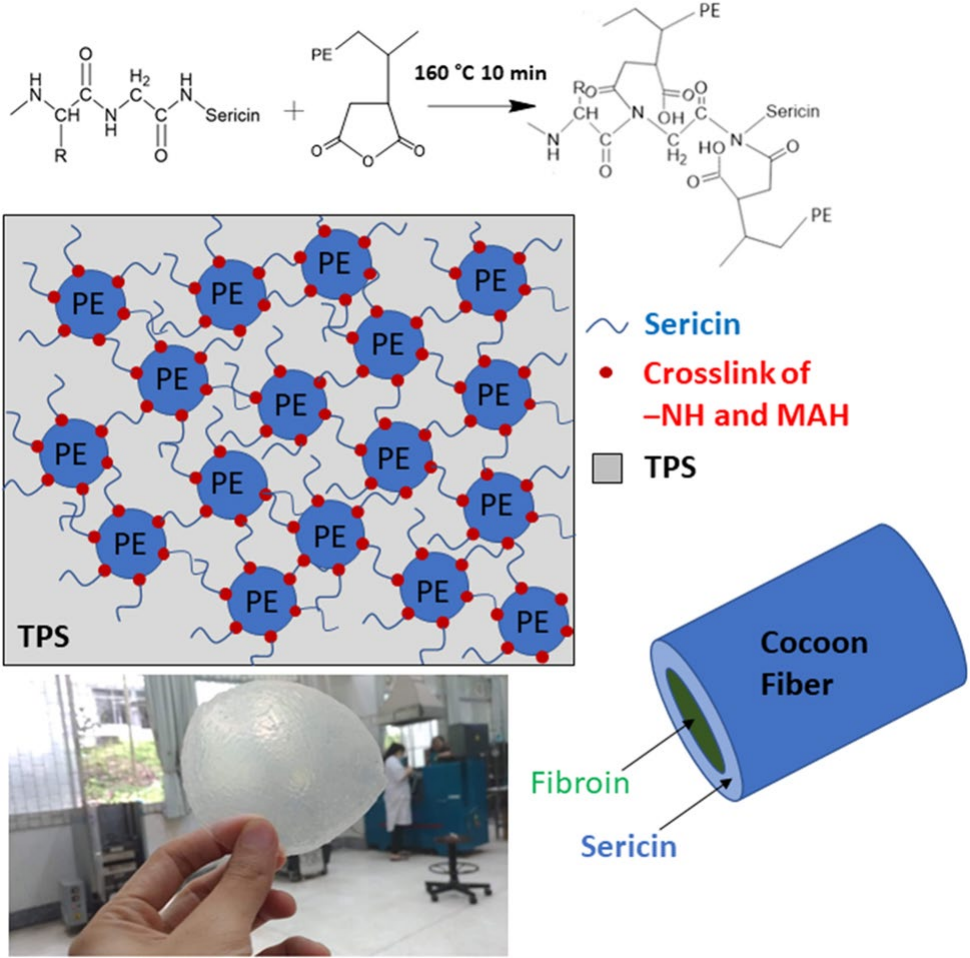
Schematic model of the TPSS/PEMAH blend through reaction mechanism of sericin/PEMAH.

Cassava starch was blended with glycerol (70:30%w/w) to prepare the TPS due to good melt–processing ability and mechanical properties^5,41^. Sericin was extracted from cocoon fiber, and sericin at 1–10 phr was incorporated into the TPS by solution mixing. TPS or TPSS (80% w/w) was melt-blended with PEMAH (20%) at 160 °C for 10 min. This study extends our investigation to develop a new compatibilizer for TPS. The ‒NH groups in sericin were suggested to react with the MAH groups of PEMAH, which improved the morphology and mechanical properties of the blends. The schematics of the sericin/PEMAH reaction mechanism with improved morphology and the TPSS5/PEMAH sample image are shown in Fig. 1.

### Tensile properties

**Figure 2:**
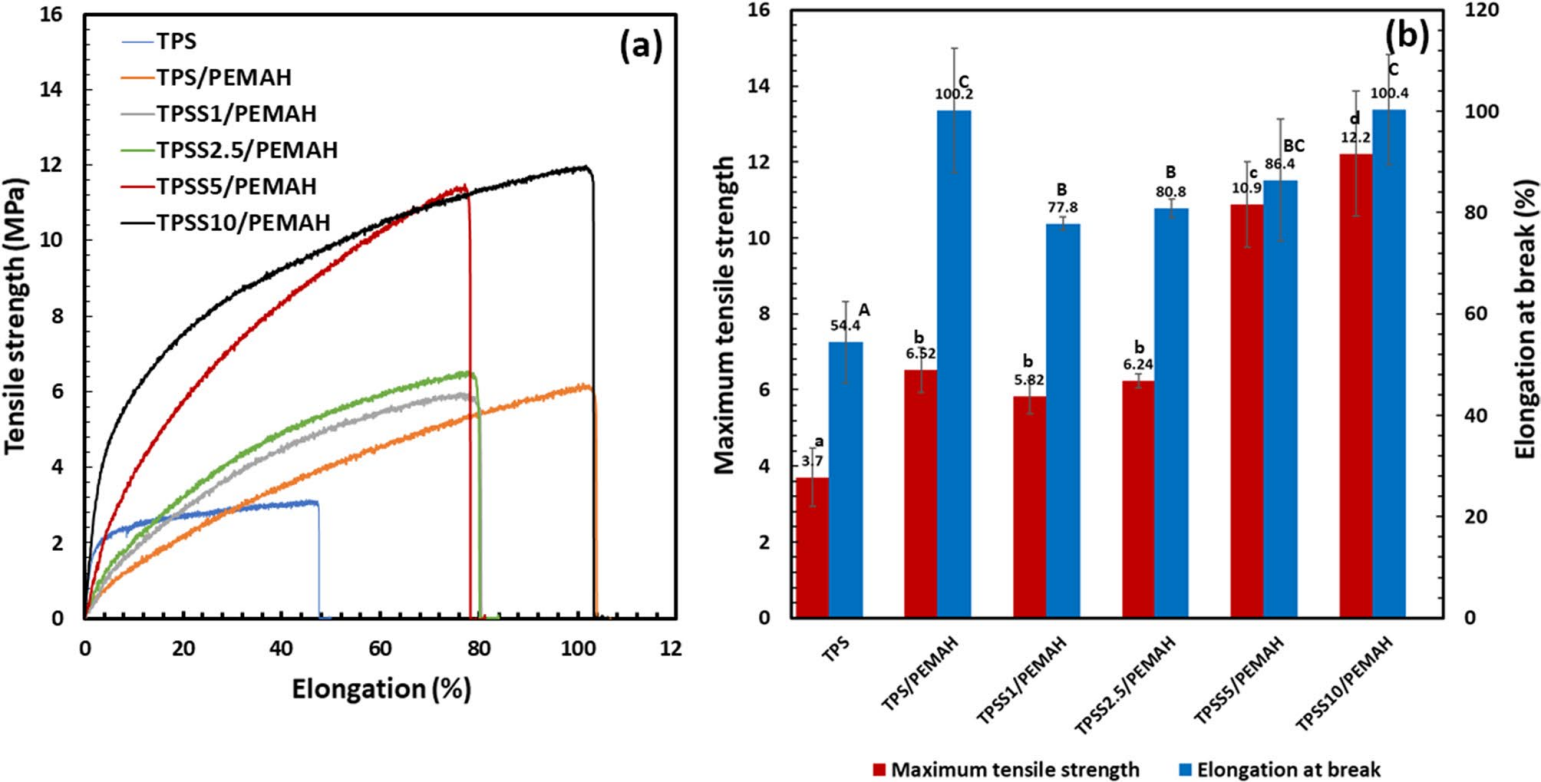
Tensile properties of thermoplastic starch (TPS) and TPS with sericin (TPSS) blending with polyethylene grafted maleic anhydride (PEMAH) (**a**) stress–strain curve and (**b**) maximum tensile strength and elongation at break (n = 5). Means with different lowercase letters of maximum tensile strength and uppercase letters of elongation at break are significantly different (P < 0.05).

The stress–strain curve is shown in Fig. 2a. The tensile strength and elongation at break of TPS were 3.7 MPa and 54.4%, respectively (Fig. 2b). TPS blending with PEMAH increased the tensile strength and elongation at break to 6.5 MPa and 100.2%, respectively, because of the reaction between ‒OH groups of TPS and MAH groups of PEMAH^42^. The interface reaction between the two polymer blends, which causes an improvement in the tensile properties, has been previously reported^6,42,43^. The tensile strength of TPS/PEMAH blend with sericin 0‒2.5 phr did not differ significantly (P < 0.05). The TPSS/PEMAH blend with sericin 1‒5 phr decreased elongation at break compared to the TPS/PEMAH because the occurred interfacial crosslink combined brittleness between TPS and PEMAH through sericin. TPSS10/PEMAH presented the highest tensile strength (12.2 MPa) and elongation at break (100%) due to high interfacial crosslinking. Tensile strength (1.4‒3.6 MPa) and elongation at break (48–101%) of TPS from cassava starch and glycerol blend have been previously reported^44,45^. Tensile strength improvement of TPS blend with PEMAH (5‒14 MPa)^41^, epoxidized natural rubber (4‒6 MPa)^5^, and poly(butylene adipate-co-terephthalate) (6‒7.5 MPa)^40^ has also been reported. Young’s modulus of TPS was 120 MPa, while the TPSS/PEMAH blend with sericin 0, 1, 2.5, 5 and 10 phr showed 20, 25, 29, 55 and 140 MPa, respectively. The tensile strength and Young’s modulus of the TPSS/PEMAH were enhanced with the sericin content because of the interfacial crosslinking^7,46^ between TPS and PEMAH and the crosslinking inside^47^ the PEMAH phase through sericin.

### Morphology

**Figure 3:**
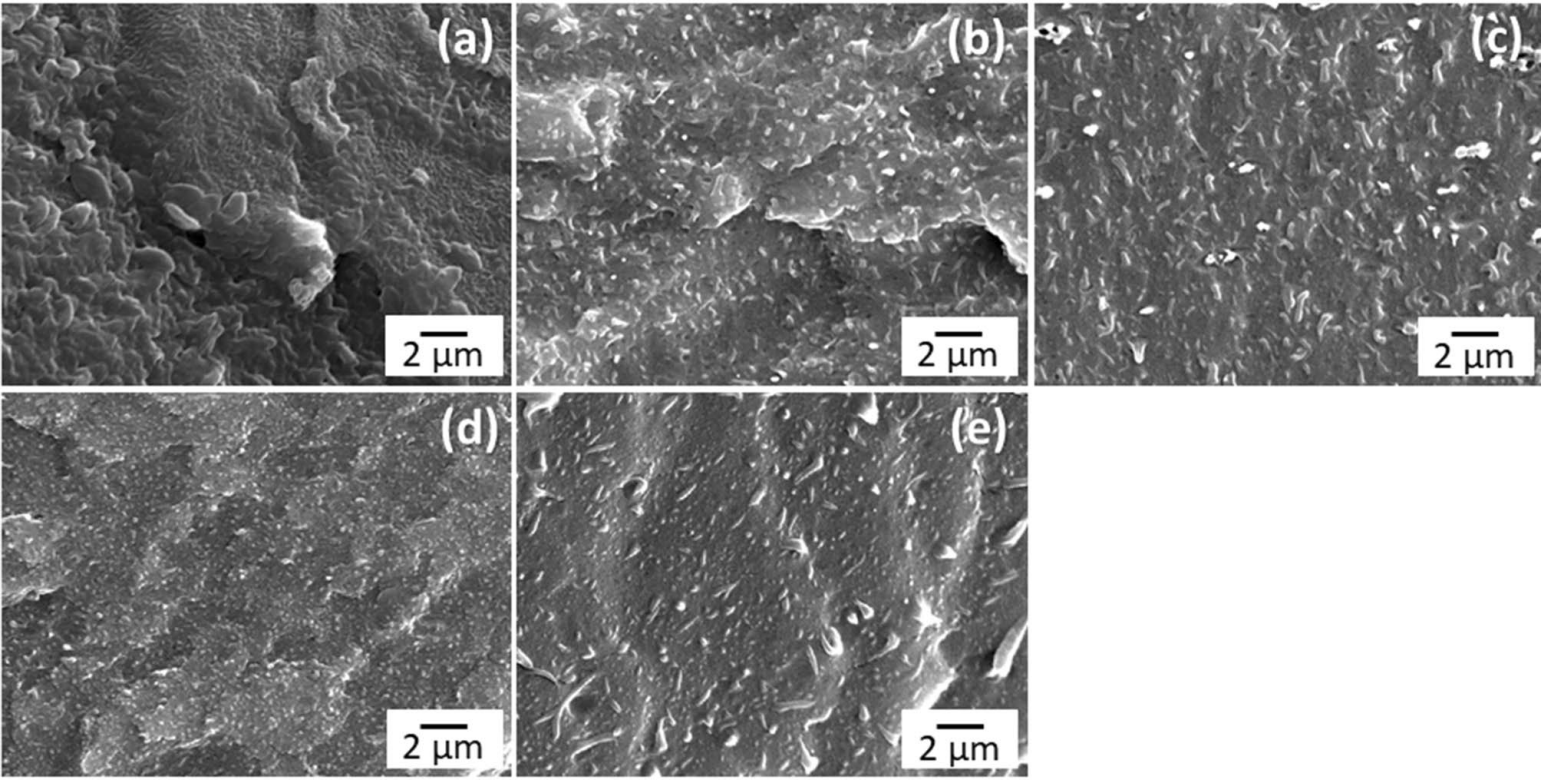
Scanning electron micrographs of (**a**) TPS/PEMAH, (**b**) TPSS1/PEMAH, (**c**) TPSS2.5/PEMAH, (**d**) TPSS5/PEMAH, and (**e**) TPSS10/PEMAH.

The morphology of TPS with sericin 0–10 phr (80% w/w) blended with PEMAH (20% w/w) is shown in Fig. 3. TPS/PEMAH showed dispersion of PEMAH particles (2 μm) in the TPS matrix. The addition of 1–5 phr sericin decreased the PEMAH particle sizes to 500 nm and 200 nm, respectively, owing to an improvement in the interfacial tension^48^ between TPS and PEMAH by sericin. Morphology of PEMAH nanoparticles in TPS matrix showed high transparency (Fig. 1). The nano-sized PEMAH dispersed in the TPS matrix provided a highly transparent material owing to the low light scattering of small particle sizes. However, the PEMAH particle sizes of TPSS10/PEMAH increased to 500 nm–1 μm compared to that of TPSS5/PEMAH because of the crosslinking inside the PEMAH phase with sericin. Such behavior of the increased particle sizes due to crosslinking inside the particle phase has been previously reported^49^. The improvement in morphology was evident from the formation of interfacial crosslinking between the interface^50^ of TPS and PEMAH via the sericin reaction, which mitigates the interfacial tension.

### Contact angle

**Figure 4:**
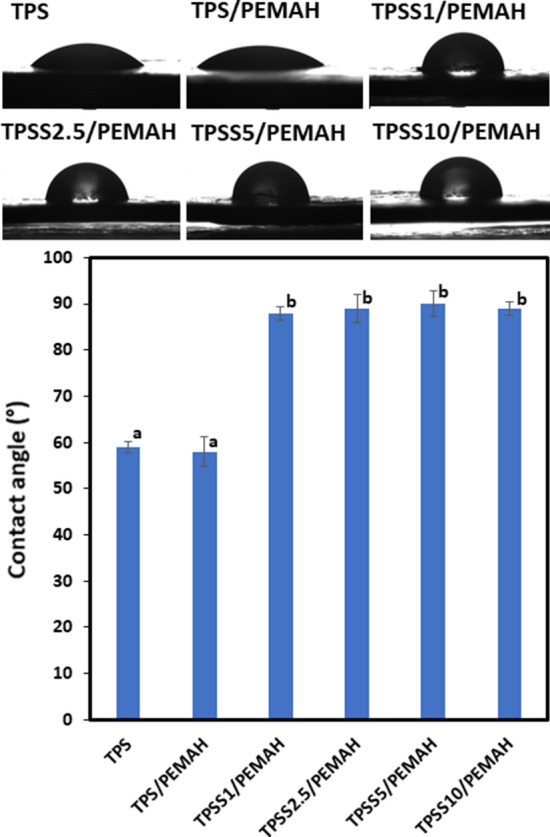
Water contact angle at 3 min of the thermoplastic starch (TPS), TPS with sericin (TPSS) 1–10 phr blending with polyethylene grafted maleic anhydride (PEMAH).

The relative interfacial tension of TPS, TPS/PEMAH and TPSS/PEMAH blend with sericin 1‒10% was evaluated using the water droplet contact. The results are shown in Fig. 4. TPS and TPS/PEMAH showed water droplet contact at 59°. Low contact angle of TPS and the TPS/PEMAH was due to hydrophilicity of cassava starch^51^ and the continuous phase of TPS. The water droplet contact angle of TPS/PEMAH blend with sericin 1‒10 did not differ significantly (P < 0.05), whereas the value increased to 88‒90° compared to TPS (59°). The improvement of water droplet contact angle of the TPS/PEMAH blend with sericin owing to hydrophobicity of polyethylene^52^ and small particles size distribution of PEMAH in TPS matrix^41^. High interfacial crosslink was indicated to improve morphology, interfacial tension, and water resistance of the TPSS/PEMAH blend, which occurred during melt mixing through sericin compatibilizer.

### Thermal properties

**Figure 5:**
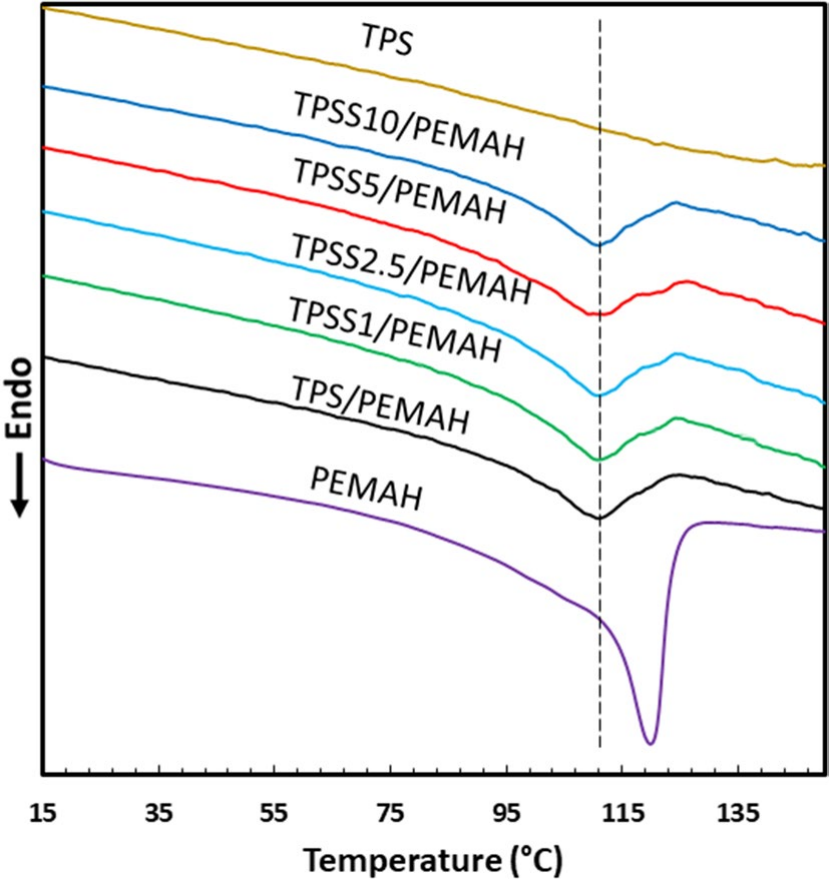
Differential scanning calorimetry curves (2nd scan) of thermoplastic starch (TPS), TPS with sericin (TPSS), and TPSS 1–10 phr blending with polyethylene grafted maleic anhydride (PEMAH).

Thermal properties were observed using differential scanning calorimetry (DSC). The DSC curves (2nd scan) are shown in Fig. 5. PEMAH showed a melting temperature (T_m_) at 121 °C, while T_m_ of TPS was not observed because glycerol induced an amorphous structure of TPS^53^. The T_m_ values of the TPS/PEMAH and TPSS/PEMAH blends were attributed to T_m_ of PEMAH at 111 °C. Moreover, the formation of small crystals at the TPS/PEMAH interface is observed. The decreasing T_m_ of PEMAH was due to the small crystal of PEMAH, which occurred at the interface of TPS/PEMAH and inside the PEMAH phase^54^. The crystal growth inside the PEMAH phase was restricted by the small sizes of the PEMAH particles, and these crystals melted at temperatures lower than pure PEMAH. The decrease in T_m_ due to the formation of small crystals has been previously reported^55^.

### Rheological properties

**Figure 6:**
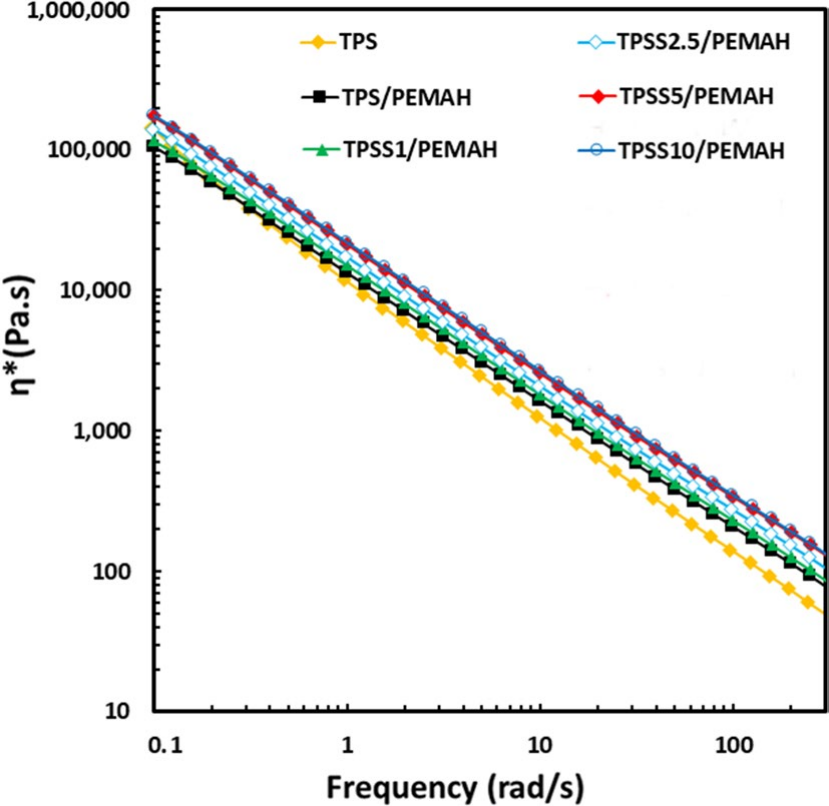
Melt-viscosity of TPS and TPSS 1–10 phr blending with polyethylene grafted maleic anhydride (PEMAH) at 160 °C.

The melt viscosities of the samples were measured at 160 °C. The η^*^ values of the samples are presented in Fig. 6. Non-Newtonian behavior of TPS and TPS blends were specified by linear viscoelasticity range of rheometer measurement. The melt viscosity of TPS was the lowest at a high frequency. TPS/PEMAH showed a higher melt viscosity than neat TPS across most of the investigated frequency ranges. The melt viscosity of TPSS1/PEMAH was higher than that of TPS and TPS/PEMAH. Additionally, the melt viscosity of TPSS5/PEMAH and TPSS10/PEMAH increased significantly because a reaction occurred during the melt-blending process^56^. The increase of melt viscosity of the TPSS/PEMAH with sericin content related to the occurred interfacial crosslinking reaction between TPS and PEMAH through sericin. This reaction extended the chain length, branch, and interfacial crosslink structures, which increased the melt viscosity of the blends^57^. The chemical reaction that influences the melt viscosity of the polymer blend has been previously reported^5,41^.

### Reaction mechanism

**Figure 7:**
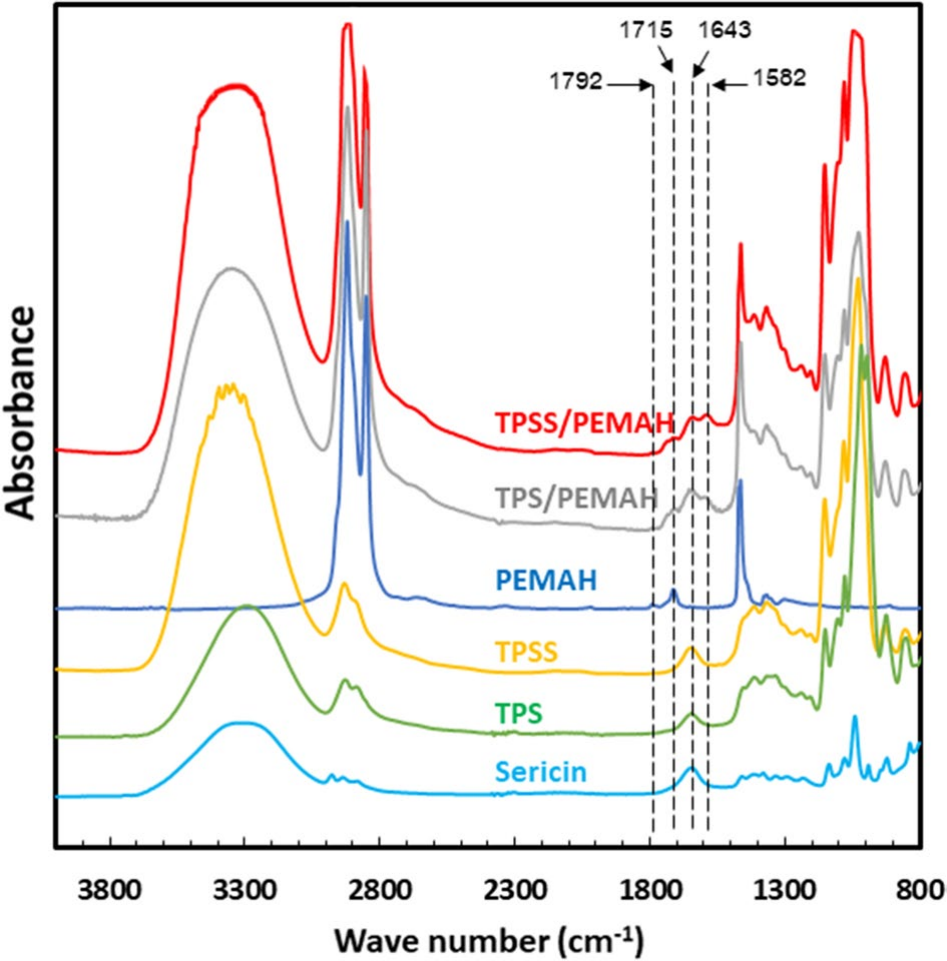
Fourier transform infrared spectra of sericin, thermoplastic starch (TPS), TPS with sericin (TPSS), polyethylene grafted maleic anhydride (PEMAH), TPS/PEMAH, and TPSS/PEMAH.

**Figure 8:**
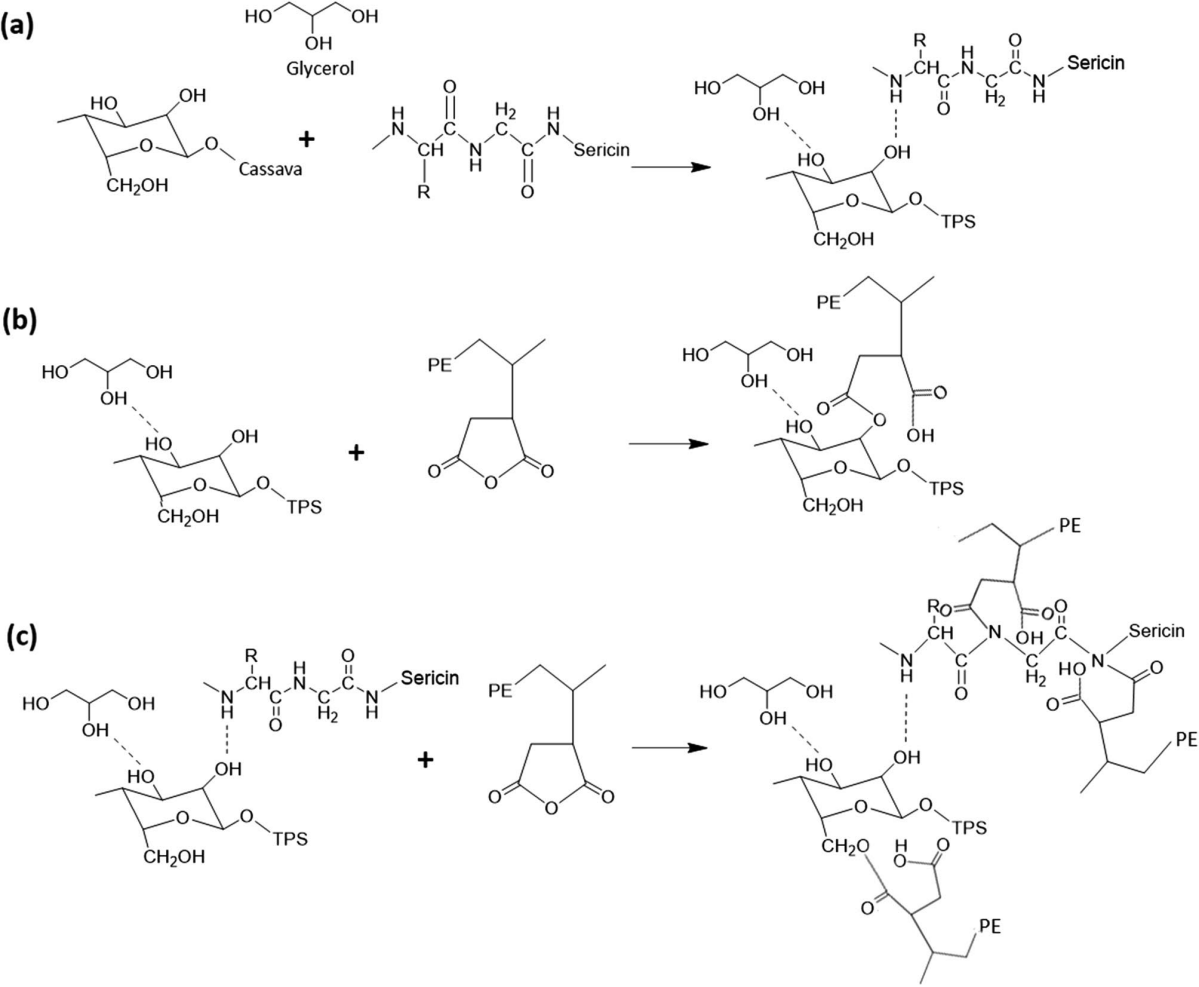
Suggested reactions and interactions (**a**) interaction of cassava starch/sericin and cassava starch/glycerol, (**b**) TPS/PEMAH reaction, and (**c**) TPSS/PEMAH reaction.

FTIR was used to investigate the reaction between sericin and PEMAH. The FTIR vibration spectra of TPS, TPSS, sericin, PEMAH, TPS/PEMAH, and TPSS/PEMAH blends at different wavelengths from 800 to 4000 cm^−1^ are shown in Fig. 7. TPS presented a peak at 1643 cm^−1^ due to ‒OH bending, while the peaks at 1016 and 929 cm^−1^ were assigned to ‒CO stretching^58^. The vibration spectra of PEMAH showed C‒H_2_, carboxylic acid, and symmetric stretching of cyclic anhydride at 1463 cm^−1^, 1715 cm^−1^ and 1792 cm^−1^, respectively. The sericin peaks at 1643 and 1240 cm^−1^ were attributed to the C=O stretching of the amides and C‒N stretching bands, respectively^59^. The blended samples were normalized using a peak at 1463 cm^−1^ of PEMAH. The TPS/PEMAH spectra showed a new peak at 1582 cm^−1^, which was not observed in the spectra of TPS, PEMAH, and sericin. This new peak indicates a new C‒O vibration from the reaction between the ‒OH groups of cassava starch and the MAH groups of PEMAH^60^. However, the TPSS/PEMAH spectra had a higher 1582 cm^−1^ intensity than in the TPS/PEMAH spectra owing to a new C‒O vibration from the reaction between the ‒NH groups of sericin and the MAH groups of PEMAH^43,61^. The suggested reactions are shown in Fig. 8. These reactions enhanced the interfacial adhesion between TPS and PEMAH, which improved the mechanical properties and morphology of the blends^62,63^. The relatively high amount of sericin (10 phr) enhanced the high crosslinking reaction inside the PEMAH phase (Fig. 8c), with an increased Young’s modulus and maximum tensile strength^64,65^ of TPSS10/PEMAH.

## Conclusions

The mechanical properties and morphology of the TPS/PEMAH blend were successfully improved using sericin as a compatibilizer. Sericin contains many ‒NH groups in its structure. The ‒NH groups of sericin formed a hydrogen bond interaction with ‒OH groups of TPS and a reaction (covalent bond) with MAH groups of PEMAH, which influenced mechanical properties, morphology, surface tension and melt viscosity of the blends. The tensile strength, elongation at break, and Young’s modulus were elevated because of the improvement in the interfacial adhesion between TPS and PEMAH and the crosslinking reaction inside the PEMAH phase via the sericin structure. The particle sizes of PEMAH were dispersed to nano-sizes owing to improvement of interfacial crosslink through sericin compatibilizer. The morphology of the nano-sized PEMAH particles in the TPS matrix enhanced surface tension and provided a highly transparent material. Moreover, the increase of interfacial crosslink and the crosslinking reaction inside the PEMAH phase also enhanced melt viscosity of the blends These crosslinks induced formation of small crystals inside the PEMAH phase, and reduced T_m_ of the TPSS/PEMAH blend compared to PEMAH. The rheological properties and FTIR results confirmed that the ‒OH groups of starch and the ‒NH groups of sericin reacted with the MAH groups of PEMAH. These reactions improved the mechanical properties, morphology, and water resistance of the TPSS/PEMAH blend. The new transparent TPS exhibits high mechanical properties, water resistance, and melt processing ability, which could be used for packaging, agriculture, and medical applications.

## Methods

### Materials

Cassava starch (Dragon fish brand), with an amylose/amylopectin content of 17/83% w/w, 11% moisture content, and a molecular weight of 1.34 × 10^8^ g/mol, was purchased from Tong Chan Registered Ordinary Partnership (Bangkok, Thailand). Glycerol was purchased from Union Science Co., Ltd. (Chiang Mai, Thailand). PEMAH (Fusabond, DuPont, Bangkok, Thailand) with a melt flow index of 1.75 g/10 min and 2% MAH content was obtained from Chemical Innovation Co., Ltd. (Bangkok, Thailand). Cocoon was kindly supplied by Chul Thai Silk Co., Ltd. (Phetchabun, Thailand).

### Sample preparation

Sericin extraction was performed using a high temperature-high pressure method^22,66^. Briefly, 45 g of the cocoon pieces (2 × 2 cm) were immersed in distilled water (1.5 L) in a 2.5 L laboratory bottle. Sericin was extracted by autoclaving at 121 °C for 30 min. The aqueous solution was filtered through filter paper and stored at 4 °C until further use. Cassava starch was mixed with glycerol (70:30% w/w) in a water bath at 80 °C for 30 min for incorporation of an additive. During premixing, sericin at 1–10 phr (part/hundred) of TPS was incorporated into the solution to modify starch. The solutions were then dried at 50 °C for 24 h to remove moisture before melt mixing. The dried samples were melt-mixed using an internal mixer (Labo Plastomill; Toyo Seiki Co. Ltd., Tokyo, Japan) to prepare the TPSS. The melt-blending of TPSS with PEMAH was also performed using the same mixer at 160 °C for 10 min. The samples were compressed into sheets at 160 °C for 3 min. The formulations of the blended samples are listed in Table 1.

**Table 1:** Composition and codes of thermoplastic starch (TPS) and thermoplastic starch with sericin blending with polyethylene grafted maleic anhydride (PEMAH).

Samples	Composition		
	TPS (w%)	PEMAH (w%)	Sericin (phr/TPS)
TPS	80	–	–
TPS/PEMAH	80	20	–
TPSS1/PEMAH	80	20	1
TPSS2.5/PEMAH	80	20	2.5
TPSS5/PEMAH	80	20	5
TPSS10/PEMAH	80	20	10

### Tensile properties

A tensile tester (Tensilion UTM-II-20; Orientec Co. Ltd., Tokyo, Japan) was used to investigate the tensile properties of the samples at a speed of 10 mm/min. The samples were compressed into sheets by compression molding at 160 °C for 3 min, followed by conditioning at 50% RH for 48 h at 25 °C. The width, length, and thickness were 2 mm × 30 mm × 1 mm. The maximum tensile strength (MPa) and elongation at break were observed for the five specimens for each composition.

### Scanning electron microscopy

The morphologies of the samples were observed using a scanning electron microscope (SM-200, Topcon Corp., Tokyo, Japan). The samples were immersed and broken into liquid nitrogen. The fracture surfaces of the samples were coated with a thin layer of gold by sputtering and observed at a voltage of 15 kV.

### Contact angle

Water droplet contact angle was observed using a drop shape analysis (DSA30E, Kruss Co. Ltd., Hamburg, Germany). The samples were prepared as films using hot compression molding at 160 °C for 3 min. Water was dropped onto the surface of the samples and recorded the images at 3 min. Five repeated samples were observed for each condition.

### Differential scanning calorimetry

Differential scanning calorimetry (DSC) (PerkinElmer Pyris Diamond DSC, Connecticut, USA) was used to observe the melting temperatures of the samples. The sample (8–10 mg) was placed in aluminum pans and put in sample holder of DSC with a pan reference. The samples were observed during the first and second scans at a heating rate of 10 °C/min. The melting temperature (T_m_) was determined from the temperature at the maximum level of the endothermic peak.

### Rheology

A rheometer (dynamic analyzer RDA II; Rheometric Scientific Corp., Delaware, USA) was used to determine the complex dynamic viscosity (η^*^) under a nitrogen atmosphere. Samples with 25 mm diameter and 1 mm thickness were compressed at 160 °C for 3 min. The experiments were performed at 160 °C with a frequency range of 0.1–1000 rad/s at a 10% strain rate (within the linear viscoelastic range of materials).

### Reaction mechanism

Fourier-transform infrared spectroscopy (FTIR-480plus; Jasco Corp., Tokyo, Japan) was used to investigate the reaction between TPS, sericin, and PEMAH. The samples were prepared as thin films by compression molding at 160 °C for 3 min. The IR spectrum was measured at 800–4000 cm^−1^ with a resolution of 4 cm^−1^.

### Statistical analysis

All results were analyzed by one-way analysis of variance (ANOVA) using SPSS software. Differences found (*P* < 0.05) were evaluated using Duncan’s test. Five replicates for each sample were used for the evaluation.
